# Early detection of malaria foci for targeted interventions in endemic southern Zambia

**DOI:** 10.1186/1475-2875-10-260

**Published:** 2011-09-12

**Authors:** Ryan G Davis, Aniset Kamanga, Carlos Castillo-Salgado, Nnenna Chime, Sungano Mharakurwa, Clive Shiff

**Affiliations:** 1 Johns Hopkins Bloomberg School of Public Health, Baltimore MD. USA; 2 The Malaria Institute at Macha, Choma, Zambia

## Abstract

**Background:**

Zambia has achieved significant reductions in the burden of malaria through a strategy of "scaling-up" effective interventions. Progress toward ultimate malaria elimination will require sustained prevention coverage and further interruption of transmission through active strategies to identify and treat asymptomatic malaria reservoirs. A surveillance system in Zambia's Southern Province has begun to implement such an approach. An early detection system could be an additional tool to identify foci of elevated incidence for targeted intervention.

**Methods:**

Based on surveillance data collected weekly from 13 rural health centres (RHCs) divided into three transmission zones, early warning thresholds were created following a technique successfully implemented in Thailand. Alert levels were graphed for all 52 weeks of a year using the mean and 95% confidence interval upper limit of a Poisson distribution of the weekly diagnosed malaria cases for every available week of historic data (beginning in Aug, 2008) at each of the sites within a zone. Annually adjusted population estimates for the RHC catchment areas served as person-time of weekly exposure. The zonal threshold levels were validated against the incidence data from each of the 13 respective RHCs.

**Results:**

Graphed threshold levels for the three zones generally conformed to observed seasonal incidence patterns. Comparing thresholds with historic weekly incidence values, the overall percentage of aberrant weeks ranged from 1.7% in Mbabala to 36.1% in Kamwanu. For most RHCs, the percentage of weeks above threshold was greater during the high transmission season and during the 2009 year compared to 2010. 39% of weeks breaching alert levels were part of a series of three or more consecutive aberrant weeks.

**Conclusions:**

The inconsistent sensitivity of the zonal threshold levels impugns the reliability of the alert system. With more years of surveillance data available, individual thresholds for each RHC could be calculated and compared to the technique outlined here. Until then, "aberrant" weeks during low transmission seasons, and during high transmission seasons at sites where the threshold level is less sensitive, could feasibly be followed up for household screening. Communities with disproportionate numbers of aberrant weeks could be reviewed for defaults in the scaling-up intervention coverage.

## Background

While *Plasmodium falciparum *is endemic throughout Zambia, the country has documented significant reductions in the burden of malaria [1,2] through the scale-up of malaria control interventions advocated by the Roll Back Malaria (RBM) Partnership [3] and Zambia's National Malaria Control Programme [4]. Zambia's implementation of the "scale-up for impact" approach has included ambitious goals as stated in RBM 2008 Global Malaria Action Plan calling for 80% population coverage rates for interventions including insecticide-treated mosquito nets (ITNs) or indoor residual spraying (IRS), intermittent preventive treatment during pregnancy (IPTp) and prompt, effective treatment of cases diagnosed by microscopy or rapid diagnostic tests (RDTs) [4]. A 2010 review of the impact of Zambia's control programme found substantial progress toward achieving these goals and concluded, "*As the infection and disease become more focal, community techniques to map malaria cases and transmission and an approach of testing and treating the remaining infected population will be required*" [2].

**Figure 1:**
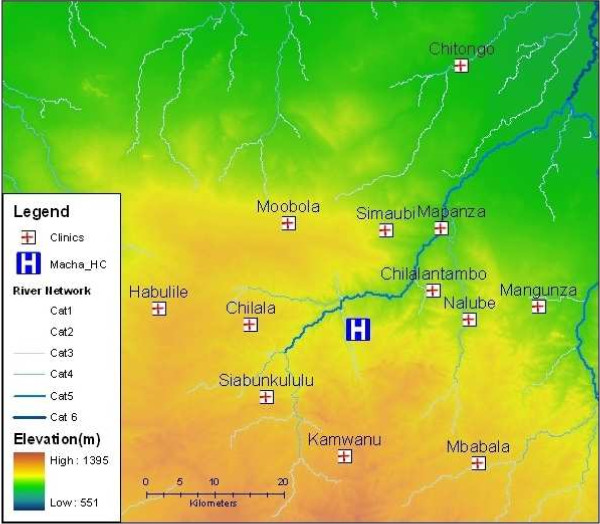
**Elevation/Contour map of the surveillance area in Choma/Namwala Districts, Southern Province, Zambia**. Rural health centres in the surveillance program are named and indicated. Drainage lines and river systems are indicated in ordinal categories. Category 1 is a simple drainage line that flows during and shortly after rain, Category 6 is a permanent large river [5].

To this end, efforts are already underway at a cluster of 13 rural health centres (RHCs) in the Choma and Namwala districts in Zambia's Southern Province (see Figure 1). Since August of 2008, a surveillance system has been established based on RDTs, for rapid and accurate diagnoses, and mobile telephones to transmit weekly data by SMS text message from the RHCs to the Malaria Institute at Macha (MIAM), located centrally [5]. The 13 RHCs serve a total population that reached approximately 158,000 people in 2010 [6]. The region is characterized as Miombo woodland, with a tropical climate consisting of three seasons: a cool, dry winter (April-August); a hot, dry season (August-November); and one hot, rainy season lasting from approximately November to April each year [7]. Although the elevation gradient is modest (See Figure 1 and Table 1), the RHC sites in the north are in or near a floodplain where the water table depth during normal seasons is only about 2-5 m below the ground [5]. The RHC sites farther south are at slightly higher elevations, away from the floodplain, and the water table is generally about 10-40 m below the ground [5]. *Anopheles arabiensis *is the predominant vector [7,8]. The discrepancy in the water table impacts the nocturnal humidity and the permanence of surface water, important for the *Anopheles *breeding and blood feeding behaviours [7,9].

**Table 1:** Patterns of malaria Rapid Diagnostic Test (RDT) diagnoses at 13 rural health centres in Southern Province, Zambia

Rural health centre	Elevation (m)	Distance from Chitongo (m)	Estimated 2010 population	% weeks missing data	% RDTs testing positive	Mean weekly incidence per 10,000 (95% CI*)		
						
						Low transmission season: weeks 23-46	First 10 weeks (47-4) of high transmission season	High transmission season: weeks 47-22
**Floodplain Zone:**						**2.15** **(1.63, 2.67)**	**3.87** **(2.26, 5.47)**	**4.63** **(3.57, 5.70)**

Chitongo	1,013	0	19,136	4.65	8.04	2.15 (1.63, 2.67)	3.87 (2.26, 5.47)	4.63 (3.57, 5.70)

**Transitional Zone:**						**0.96** **(0.66, 1.26)**	**1.58** **(1.11, 2.05)**	**2.80** **(2.30, 3.30)**

Mapanza	1,059	23,842	22,710	3.10	8.07	0.32 (0.20, 0.43)	1.36 (0.29, 2.44)	2.56 (1.73, 3.40)

Simaubi	1,117	26,414	9,794	33.33	9.16	0.14 (0.01, 0.27)	2.41 (1.25, 3.56)	3.57 (2.51, 4.63)

Chilalantambo	1,100	33,062	3,411	11.63	11.73	1.99 (1.23, 2.76)	1.22 (0.46, 1.99)	3.03 (1.73, 4.33)

Nalube	1,110	36,944	3,411	12.40	11.65	1.18 (0.46, 1.89)	1.57 (0.81, 2.32)	2.25 (1.46, 3.03)

**Heartland Zone:**						**0.22** **(0.14, 0.29)**	**0.70** **(0.42, 0.99)**	**3.25** **(2.53, 3.98)**

Mangunza	1,087	36,869	12,729	22.48	16.11	0.35 (0.05, 0.65)	0.41 (0.17, 0.64)	2.36 (1.47, 3.26)

Moobola	1,165	34,127	18,731	23.26	2.60	0.09 (0.02, 0.15)	0.51 (0.23, 0.78)	1.02 (0.73, 1.32)

Macha	1,136	41,245	19,950	6.98	7.15	0.25 (0.12, 0.39)	0.80 (0.34, 1.27)	1.68 (1.19, 2.17)

Chilala	1,187	48,677	12,565	24.03	10.97	0.08 (-0.02, 0.18)	0.14 (-0.07, 0.35)	1.47 (0.80, 2.15)

Siabunkululu	1,190	56,024	12,064	21.71	9.84	0.25 (0.08, 0.41)	0.25 (0.00, 0.50)	2.86 (1.70, 4.02)

Habulile	1,210	56,465	9,424	6.20	15.17	0.34 (0.09, 0.59)	1.24 (0.21, 2.29)	3.88 (2.67, 5.06)

Mbabala	1,204	57,941	13,023	7.75	2.31	0.01 (-0.01, 0.03)	0.07 (-0.01, 0.15)	0.56 (0.21, 0.91)

Kamwanu	1,273	59,384	1,333	35.66	5.29	0.48 (-0.09, 1.04)	2.25 (0.26, 4.24)	13.12 (8.07, 18.17)

* bootstrap confidence intervals with 10,000 replications

**Figure 2:**
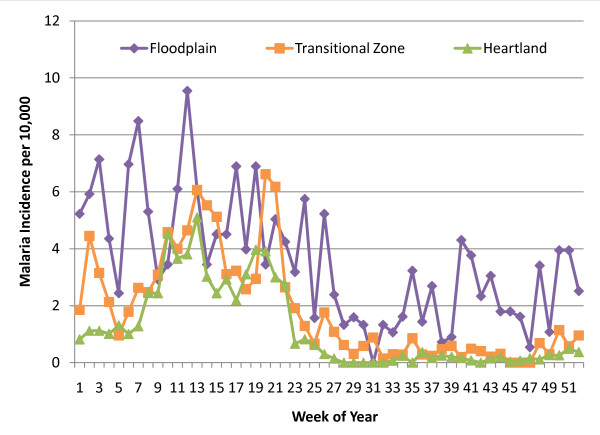
**Weekly malaria incidence (RDT-confirmed) throughout an average year (January through December) at rural health centres in the Choma and Namwala Districts in Southern Province, Zambia**. * The centres were separated into three zones based on locality, elevation and incidence patterns (See Figure 1 and Table 1).

The RDT-diagnosed malaria incidence reflects the impact of these geographic and seasonal conditions (See Figure 2). In a typical 52-week year, a high transmission season is observed beginning in week 47 and continuing through week 22 of the subsequent year. This corresponds only approximately with the November to April rainy season. The weeks of elevated incidence start a few weeks later than the beginning of the rainy season and end a few weeks later than the end of the rainy season. This lag may be attributed to the *Plasmodium falciparum *incubation delay, a diagnostic delay, the persistence of surface water in the weeks after the rainy season and the *Anopheles *behaviour (the vectors gradually increase in number during the rainy season, with the development of breeding habitats, and peak in March and April [7]).

Exploratory analysis reveals different incidence patterns at rural health centres grouped by locality and elevation (See Figure 2 and Table 1). Chitongo is located in the floodplain and incident malaria cases are regularly diagnosed throughout the year. Four RHCs near the floodplain, and serving some homesteads in the floodplain, have minimal incidence during the low transmission season (weeks 23-46). Incident cases are rare during the low transmission season at the eight RHCs in the southern zone that has been termed the "heartland" [5]. The incidence of diagnosed malaria rises more rapidly at the RHCs in the transitional zone between the floodplain and the heartland as compared to those sites within the heartland. During the 10 weeks at the beginning of the high transmission season (weeks 47-4), the mean incidence of malaria diagnoses is 1.58 (1.11, 2.05) per 10,000 people at sites in the transitional zone, while it is only 0.70 (0.42, 0.99) per 10,000 people at sites in the heartland. However, the overall high transmission season mean incidence is comparable in all three zones (See Table 1).

During periods when mosquito populations are minimal, asymptomatic infection serves as a reservoir for the parasite population and a source of efficient transmission when mosquito populations expand [10,11]. Efforts to move beyond the advances of the scale-up phase of the Zambia malaria control program, ultimately with an eye toward elimination, will need to target these asymptomatic infections. The transition from burden reduction to transmission interruption requires active methods to supplement passive case detection for identification and elimination of malaria foci [12]. Planning for such a programme requires a realistic accounting of logistic and financial feasibility. "Proactive" or "aggressive active case detection" is resource intensive. Each positive diagnosis in a proactive control strategy implemented in the Brazilian Yanomami area was estimated to cost 2.3 times more than a passive diagnosis [13]. Population mobility and testing fatigue are also concerns [12]. Presently, such a programme is likely infeasible in Zambia, given the constrained resources and the commitment required to sustain current gains and advance toward the "scaling-up for impact" 80% coverage goal [4]. "Reactive" case detection, in contrast, is a more modest undertaking, triggered when a case is identified by passive case detection, and involves screening around the index case. As such, surveillance becomes useful beyond just the monitoring and evaluation of interventions but as a control intervention in itself.

The programme in southern Zambia, making use of RDTs and mobile phones, facilitates timely and accurate dissemination of local surveillance data that could be used for reactive case detection. A small pilot study at four of the RHCs (Nalube, Chilalantambo, Mapanza, and Chitongo) supported the feasibility of reactive case detection methods to identify asymptomatic infections during the dry season. It was hypothesized that these silent malaria reservoirs would be vulnerable to targeted control measures during periods of low transmission [14]. All of the malaria diagnoses from June to August of 2009 at these four RHCs were followed up with a visit to their homestead. All consenting residents completed a questionnaire, were tested for malaria, and those testing positive by RDT received treatment. Prevalence, as determined by PCR diagnosis, was statistically significantly higher (p = 0.006) among homesteads identified through passive case detection (8.0%) as compared to a control group of randomly selected households (0.7%). While a gametocyte prevalence of 2.3% was found in the case population and none in the control group, this difference was not statistically significant (p = 0.145). A larger sample size and data from multiple seasons could further validate this approach to identify asymptomatic malaria foci [14].

In seasonally endemic regions like southern Zambia, the term "epidemic" is essentially used to describe any occurrence of malaria cases in excess of normal [15]. However, there is a lack of precision regarding what should be expected as "normal" on any given week at any specific RHC in an environment with such seasonal and geographically driven incidence variability. An early detection system to identify and target foci of aberrant case burdens (especially during the transitional weeks 47-4) before they spread to adjacent communities could build on the success observed during the dry season when acute exacerbations of incidence are more apparent.

The RBM programme supports the establishment of surveillance and early detection systems to prevent or contain malaria epidemics: "*Timely detection of cases of illness in a community or region, above the normally expected level, is vital to ensure that health authorities and policy-makers are aware of the serious and immediate threat before them and to help them make decisions on effective control measure*s" [16]. A RBM framework for public health workers in Africa suggests a variety of simple methods to develop temporal aberration detection thresholds in resource-constrained settings [16]. See Table 2 for a summary of alert threshold calculation techniques.

**Table 2:** Comparison of alert threshold development techniques

Technique	**Methods to calculate threshold **[16,20]	Advantages	Disadvantages
**WHO **[17]	Upper third quartile of monthly case numbers from preceding 5 years	• Calculation does not require a computer • Not skewed by epidemic years	• Requires 5 years of historic data • Limited utility for local public health response when calculated at the monthly and district-wide level

**Cullen **[18]	Monthly mean number of cases + 2 standard deviations from 5 years of historic data where "epidemic years" have been excluded	• Simple calculation	• Requires 5 years of historic data • Limited utility for local public health response when calculated at the monthly and district-wide level • Exclusion of "epidemic years" is arbitrarily defined

**C-sum **[19]	Mean number of cases for a given month, the preceding month and the subsequent month from the past 5 years plus 2 standard deviations (note: the same technique has been applied to weekly data for a variety of diseases including malaria [23]).	• Smooths fluctuations due to irregular reporting rather than disease incidence by providing a larger 15 historic months sample size.	• Requires 5 years of historic data • Limited utility for local public health response when calculated at the monthly and district-wide level

**Poisson **[20]	Upper 95% confidence interval limit of Poisson distribution based on weekly case numbers from past 2 or more years of historic data at sites grouped by transmission zones and adjusted by population of catchment areas.	• Granular weekly and local thresholds better reflect the seasonal and geographic variations and allow for more agile public health responses • Does not require as many years of historic data • A larger historic sample size is obtained by grouping sites with similar observed patterns of transmission	• Greater influence of "epidemic years" on mean and threshold calculations because fewer years of historic data are used • Questionable applicability of Poisson assumptions • Zonal thresholds introduce an aggregation bias with inconsistent sensitivities between RHCs within a zone

One very basic method advocated by the WHO [17] does not require access to a computer. An alert threshold is set at the upper third quartile of the previous five years of monthly case numbers for a given location (ie the second highest value for each month among the five years). A method proposed by Cullen *et al *[18] uses the previous five years of data to determine the monthly mean number of cases and sets the epidemic threshold at the mean plus two standard deviations. Because abnormal years have a greater influence on this method than the WHO technique, "epidemic" years are arbitrarily excluded. The Centers for Disease Control developed the cumulative sum (c-sum) method [19] to increase the historical sample size and reduce the impact of aberrant months on the threshold calculation. The historical baseline is calculated as the average of the reported number of cases for the preceding month, the corresponding month and the following month, for the previous five years. The mean plus two times the standard deviation of these 15 correlated observations is used to calculate a threshold value.

The Cullen *et al *technique (using the five-year historic monthly mean plus two standard deviations) was applied to cases of *Plasmodium vivax *in northern Thailand during the 1980s [20]. A 2006 paper out of the Thailand Ministry of Public Health Bureau of Vector Borne Disease responded to the Cullen method suggesting, "*this reporting mechanism is not timely enough to detect the occurrence of a malaria epidemic which usually occurs at the district level over a short period of time*" [20]. As an alternative, they proposed and tested an early detection method employing a *Poisson *distribution in the malaria endemic Kanchanaburi Province. The province was divided in two zones with different transmission patterns. Following a detection system described by Delacollette in 2001 [21], separate alert thresholds were calculated by determining a 95% confidence interval around the weekly mean case numbers during the two years of 2000 and 2001 at nine Vector Borne Disease Control Units divided between the two transmission zones [20]. Validating the threshold levels against a historic "epidemic year" and prospectively testing the threshold for the year of 2002, the authors conclude that the Poisson distribution offers "*an effective alternative method for the development of an early detection system*" [20].

Here this Poisson technique was applied to the Southern Province Zambia surveillance data, calculating weekly thresholds for each of the three zones (floodplain, transitional zone, and heartland) and the system was validated against historic incidence data at the RHC level.

## Methods

Separate early detection thresholds were developed for three zones comprised of 13 RHCs in Southern Province, Zambia. The RHCs were separated into zones based on elevation, locality and observed incidence patterns (See Figure 1 and Table 1). The weekly number of RDT-diagnosed malaria cases were obtained from each of the 13 sites for 129 weeks beginning in August, 2008. Population estimates from a 2000 census for the catchment area of each of the RHCs [6] were adjusted with an estimated 3.0% annual growth rate [4,6].

**Figure 3:**
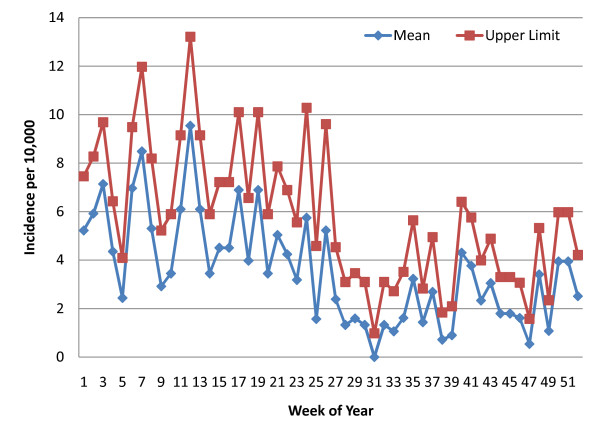
**Weekly Poisson distribution of malaria incidence (RDT-confirmed), Floodplain (1 rural health centre)**.

**Figure 4:**
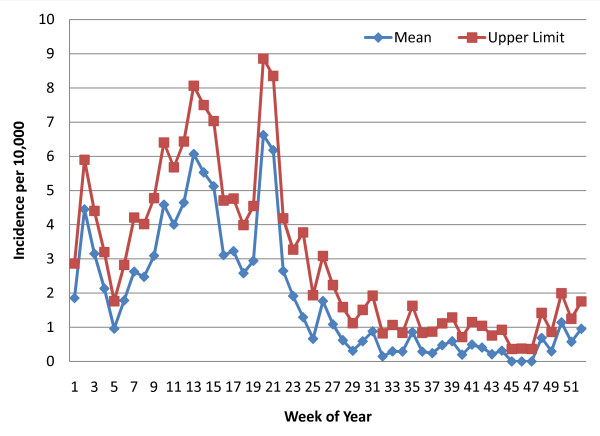
**Weekly Poisson distribution of malaria incidence (RDT-confirmed), Transitional Zone (4 rural health centres)**.

**Figure 5:**
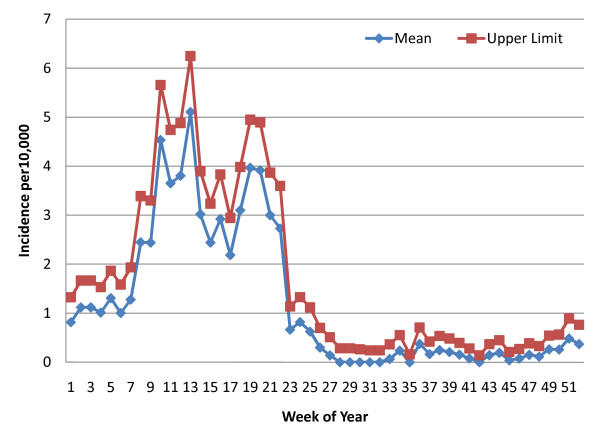
**Weekly Poisson distribution of malaria incidence (RDT-confirmed), Heartland (8 rural health centres)**.

Using STATA software [22], weekly mean incidence and 95% confidence intervals were calculated with a Poisson distribution based on the number of diagnosed malaria cases and population estimates as the weekly person-time of exposure. Data were available from week 34 of 2008 through week six of 2011. However, there were many lacunae (See Table 1) due to occasional RDT stock depletion, staff shortages or delayed SMS reporting to the central register at MIAM. For any given week of the 52 weeks in a year, the mean and confidence interval calculation included the number of cases for that week and the population of the catchment area (adjusted annually) for every year with data available and at each of the sites within the zone (the floodplain zone calculation was based on the Chitongo data alone). Weeks where data were missing from a RHC were excluded from the calculations. The upper limit of this confidence interval was considered the alert threshold for early detection at each of the RHCs within the zone. Line graphs of the weekly means and the upper 95% confidence interval limits for each of the three zones were constructed (See Figures 3, 4 and 5).

The zone threshold levels were compared to the weekly incidence of malaria diagnosis at each of the 13 respective RHCs (See Table 3). The overall percentage of weeks exceeding the threshold out of all weeks with data available was calculated, and stratified by transmission season. The year-round percentages were also calculated for the 2009 and 2010 years specifically (when the surveillance system was in place throughout the entire year). In an effort to determine if a breach of the threshold was predictive of continued elevated incidence, the percent of those weeks above the threshold which were part of a string of three or more consecutive aberrant weeks was also calculated.

**Table 3:** Comparison of weeks exceeding threshold levels at each of the 13 rural health centres

Rural health centre	Weeks above threshold				
	
	2009	2010	Overall (August 2008 through February 2011)		
			
			Low transmission seasons	High transmission seasons	Total
**Floodplain Zone:**					

Chitongo	9 (19.6%)	1 (1.9%)	5 (9.1%)	13 (19.1%)	18 (14.6%)

**Transitional Zone:**					

Mapanza	12 (24.0%)	2 (4.0%)	2 (3.5%)	15 (22.1%)	17 (13.6%)

Simaubi	9 (52.9%)	6 (12.0%)	1 (2.7%)	17 (34.7%)	18 (20.9%)

Chilalantambo	17 (36.2%)	16 (32.0%)	21 (38.9%)	16 (27.1%)	37 (32.7%)

Nalube	13 (27.7%)	7 (15.6%)	8 (15.1%)	13 (21.0%)	21 (18.3%)

**Heartland Zone:**					

Mangunza	2 (7.1%)	19 (40.0%)	7 (18.9%)	17 (27.0%)	24 (24.0%)

Moobola	7 (24.1%)	2 (3.9%)	1 (3.1%)	10 (15.6%)	11 (11.5%)

Macha	11 (21.2%)	9 (17.3%)	13 (21.3%)	13 (21.0%)	26 (21.1%)

Chilala	8 (20.0%)	1 (2.5%)	2 (3.8%)	8 (17.4%)	10 (10.2%)

Siabunkululu	13 (29.5%)	6 (15.4%)	7 (12.1%)	15 (34.9%)	21 (20.8%)

Habulile	18 (35.3%)	13 (28.9%)	7 (12.3%)	30 (47.9%)	37 (30.6%)

Mbabala	0 (0.0%)	2 (4.1%)	0 (0.0%)	2 (3.4%)	2 (1.7%)

Kamwanu	15 (42.9%)	15 (35.7%)	2 (6.3%)	28 (54.9%)	30 (36.1%)

* Figures are number of weeks above the threshold in the surveillance period. In parenthesis is the percentage of weeks above the threshold out of those weeks with data available.

## Results

In Figures 3, 4, and 5, the upper limit of the confidence interval is considered the alert level for early detection of impending aberrant elevations in malaria incidence. An abnormal incidence was detected if the number rose above the upper limit for that week. The threshold values follow the seasonal pattern of the three transmission zones, and the "detection area" between the weekly mean and the upper limit of the confidence interval is greater for weeks in the high transmission season than those the low transmission season for each of the three zones. This allows for more cases and greater variability during the high transmission season before an alert is triggered.

The percentage of aberrant or "epidemic" weeks during the entire surveillance period ranged from 1.7% in Mbabala to 36.1% in Kamwanu (see Table 3). The percentage of weeks above the threshold was generally greater during the high transmission season (except at the Simaubi and Macha RHCs), and the 2009 percentages were greater than that of 2010 except at the Mangunza and Mbabala RHCs. 39.0% of all weeks breaching the alert level were part of a series of three or more consecutive aberrant weeks. This rises to 45.9% of aberrant weeks when only considering weeks in high transmission seasons.

**Figure 6:**
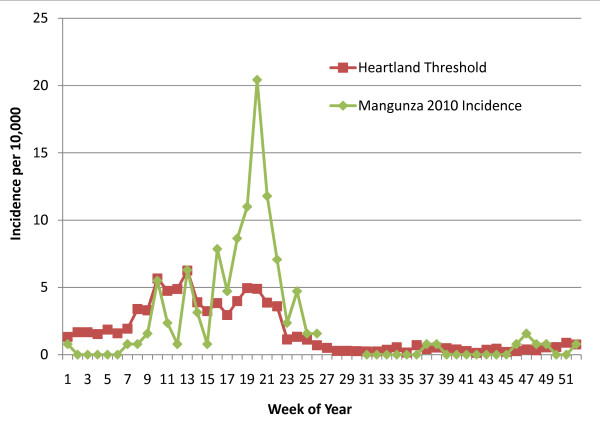
**2010 weekly malaria incidence (RDT-confirmed) in Mangunza as compared to the Heartland Zone weekly threshold level**.

A good example of early detection is found at the Manguza RHC during the 2010 year (See Figure 6). The weekly incidence first breaches the threshold level at week 13 but dips back below the threshold during weeks 14 and 15. On week 16, the incidence again exceeds the upper limit of the Poisson distribution. The incidence remains aberrantly elevated for 11 weeks, peaking at the week of May 10^th ^through the 16^th ^(this week saw 26 cases for an incidence of 20.42 diagnosed cases per 10,000 people in the Mangunza catchment area). Here, the threshold detected the abnormally elevated incidence four weeks before its peak and at a time when it might not have been otherwise anticipated (occurring after the end of the typical rainy season, but still within the observed high transmission season). Throughout the 2010 low transmission season at the Mangunza RHC, all seven weeks with one or more incident cases breached the threshold (See Figure 6). At the end of the low transmission season and the start of the subsequent high transmission season, another consecutive series of 4 weeks (week 46 through week 49) persisted above the threshold level. Each of these weeks had only one or two incident cases.

## Discussion

The Poisson technique was selected (instead of alternative methods recommended by the RBM programme [16]) based on the reported successful implementation in several endemic areas of Thailand [20], because it recognizes the importance of granular weekly and local level thresholds to allow for an agile public health response, and because it does not require five years of historic data (See Table 2). Konchom *et al *used two years of data from nine sites. Less than three years of data were currently available from the 13 RHCs in Southern Province, Zambia. The applicability of the Poisson distribution assumptions to this context is questionable. The probability of a malaria diagnosis during a time interval is not independent of the probability of a diagnosis in other intervals. However, the diagnosis of malaria during any given week is a relatively rare event for any given member of the population (See Table 1), and Konchom *et al *suggest that "*Malaria data fit the assumptions of the Poisson distribution well enough to generate an early detection system for malaria epidemics, and provide a better fit [than alternative techniques] for monitoring the malaria situation within a limited time period*" [20].

Threshold levels were calculated for zones rather than for specific RHCs because similar incidence patterns were observed in clusters of clinics (See Figure 2) and to achieve a higher sample size. Given the frequent lapses in the data collection and reporting (See Table 1), a few weeks of the year did not have any historic weeks on which to base a mean and confidence interval calculation at a given RHC, and many weeks had only one historic week of data. Neighboring RHCs with similar observed incidence patterns were grouped together in the hopes of obtaining less arbitrary threshold levels.

However, this grouping introduces an aggregation bias, and RHCs with more weeks of missing data (See Table 1) contributed less to the threshold calculations. Clearly the threshold values were more sensitive for some RHCs than for others (See Table 3). The frequent alert signals at some of the RHCs (eg Kamwanu) makes the usefulness of the threshold to identify foci of increasing incidence questionable for these sites. Kamwanu is located well within the heartland zone. It is the RHC at the highest elevation, farthest from the floodplain, and its incidence pattern follows the timing of the transition from low to high transmission season seen at other heartland sites. However, it is clear from the overall high transmission season mean weekly incidence (noted in Table 1) that the magnitude of the rise in incidence during the high transmission period has been significantly higher than the other RHCs within its zone. Discrepancies in health-seeking behaviour, population mobility, rate of population growth since the 2000 census, access and adherence to scale-up interventions, local *Anopheles *larval breeding conditions [9], and clinical malaria case definitions are among the factors that could contribute to the variable sensitivity of threshold levels at different RHCs within the transitional and heartland zones.

From Table 1, it is clear that the role of RDTs in case management differed between the various RHCs. Rural health authorities in Zambia are instructed to test only patients with symptoms suggestive of malaria including fever, but more specific clinical case definitions could standardize the use of RDTs [5]. Only the central Macha site adhered to the RDT results strictly. Other RHCs often treated for malaria based on the clinical presentation despite a negative RDT. The inconsistent percentage of positive tests compared to RDTs used makes it tempting to attribute the abnormally high incidence at Kamwanu and other RHCs to liberal case definitions that led to widespread RDT use. Kamwanu has a relatively low percentage of positive tests. However, the site with the lowest percentage of positive RDTs (suggesting the least specific clinical case definition) was also the site with the fewest weeks breaching the alert threshold (Mbabala) and there is no clear association among the incidence and RDT use patterns of the other RHCs. It is important to note that a positive RDT is a clear diagnostic result (within the limits of the test's sensitivity and specificity), and although use patterns were variable, the surveillance numbers used in the development of the thresholds here represent confirmed diagnoses, more reliable than the clinical cases sometimes used for early detection systems [15,17].

The percentage of aberrant weeks that are part of three or more weeks of consecutively elevated incidence, suggest that threshold breaches are often indicative of persistently elevated levels during the years included in the threshold calculation. The thresholds should be further tested against subsequent years. In the future, as the MAIM surveillance system continues and more years of data are available, greater specificity in detecting impending weeks of consecutive incidence elevation could likely be achieved by developing individual threshold levels specific to each RHC. When five years of historical data are obtained, alternative threshold development methods [17] could also be attempted and compared with the Poisson technique outlined here. A 2002 paper by Hay *et al *compared the application of the WHO, Cullen and c-sum techniques in the context of the seasonally-malaria-endemic highlands of western Kenya and found significant discrepancies in the sensitivity of the different techniques [15].

Hay *et al *suggest the use of a moving average to adjust threshold levels for secular trends when cases are seen to be consistently increasing or decreasing over the years used in the calculation [15]. More years of surveillance are needed to confirm a sustained trend, but Table 3 shows that the majority of RHCs had fewer weeks above the threshold in 2010 than in 2009. All of the data came from years after the roll out of the scaling-up interventions [2], but coverage may still be improving. Indeed, breeches of the threshold levels could suggest communities that should be reviewed for shortfalls in the scaling-up coverage.

The calculated threshold levels were below 1 per 10,000 in the heartland and below 2 per 10,000 in the transitional zone throughout most of the low transmission season. Although the percentage of aberrant weeks was generally lower during the low transmission season, almost any cases during this season triggered the alert thresholds for these zones. This supports the strategy of the reactive case detection pilot study that followed up on all cases detected during the dry season and successfully identified reservoirs of asymptomatic infection [14]. It remains to be seen if the more frequent alerts during high transmission seasons could also be used for reactive case detection to identify asymptomatic foci, targeting interventions to curtail further increases in incidence and interrupt the transmission to nearby communities.

## Conclusion

In the Choma and Namwala Districts of Southern Province, Zambia, a novel surveillance system based on RDT diagnosis of all suspected malaria cases and SMS mobile phone transmission of weekly data to a central registry has been implemented [5] as a tool to advance malaria control beyond the burden reduction achieved by scaling-up interventions [2]. The development and implementation of an appropriate early warning system could improve the capacity of local public health officers for decision-making, prevention and malaria control. It is reasonable to hypothesize that foci of elevated malaria diagnoses are associated with and driven by pockets of asymptomatic infections. Reactive case detection strategies, triggered by weeks of aberrant incidence, could be useful for identification of these silent reservoirs to limit the duration of "epidemic" periods and to interrupt the spread to adjacent communities.

The inconsistent reliability and questionable validity of the threshold calculations outlined here as applied to the various RHCs suggest that additional years of data may be required to refine the levels for specific sites before they could be considered operationally robust (especially for the high transmission seasons). Aberrant weeks during low transmission seasons, and during high transmission seasons at sites where the threshold level is less sensitive, could feasibly be followed up for screening of family members at their homestead. Communities with a disproportionate number of weeks above the threshold level could be assessed for defaults in the scaling-up intervention coverage. The review of this alert system supports the general utility of timely, local surveillance. Similar systems for RHC-level data collection should be implemented elsewhere in Zambia to facilitate ongoing control measures and to build the records necessary for early detection threshold development.

## Competing interests

The authors declare that they have no competing interests.

## Authors' contributions

RGD wrote the paper and performed the major part of the analysis based on data supervised and collected by AK in Zambia. NC performed initial analysis based on AK's data set. CC-S reviewed the epidemiological analysis and interpretation. SM provided overall supervision at MIAM. CS oversaw the analysis and developed the concept. All authors read and approved the final manuscript.
